# Improvement of Rat Survival and Liver Mitochondrial Function in Biliary Obstruction After Treatment With Sodium Thiosulfate

**DOI:** 10.1155/1995/43679

**Published:** 1995

**Authors:** B. Myslovaty, S. Kyzer, H. Levinsky, C. Chaimoff

**Affiliations:** Department of Surgery “A”. Hasharon Hospital Golda Medical Center Petah-Tiqva Tel Aviv University Medical School Israel

## Abstract

The exact cause of liver failure occurring after long standing biliary obstruction is not known. Impairment of hepatic mitochondrial respiration was postulated in some studies. Sodium thiosulphate (STS) is known to have a protective effect on liver function during administration of hepatotoxic chemotherapy. In the present experimental study the effect of treatment with STS in the presence of obstructive jaundice was studied by determination of the survival rate of rats subjected to biliary obstruction and by polarographic determination of the hepatic mitochondrial function. Treatment with STS was found to result in a significant improvement in rats' survival rate (p < 0.05). Polarography demonstrated significant preservation of mitochondrial respiratory capacity after treatment with STS. The results of the present study show that the deterioration in liver function in the presence of biliary obstruction is probably caused by impairment of mitochondrial respiration. This may be preserved by treatment with STS. The exact explanation of its effect is not yet clear.


*HPB Surgery*, 1995, Vol. 8, pp. 249–252
Reprints available directly from the publisher
Photocopying permitted by license only

© 1995 OPA (Overseas Publishers Association)
Amsterdam B.V. Published in The Netherlands
by Harwood Academic Publishers GmbH
Printed in Malaysia

### Improvement of Rat Survival and Liver Mitochondrial Function in Biliary Obstruction After Treatment With Sodium Thiosulfate

B. MYSLOVATY, S. KYZER, H. LEVINSKY and C. CHAIMOFF

Department of Surgery "A". Hasharon Hospital, Golda Medical Center,
Petah-Tiqva, Tel Aviv University Medical School, Israel

*(Received November 17, 1993)*

The exact cause of liver failure occurring after long standing biliary obstruction is not known. Impairment of hepatic mitochondrial respiration was postulated in some studies. Sodium thiosulphate (STS) is known to have a protective effect on liver function during administration of hepatotoxic chemotherapy. In the present experimental study the effect of treatment with STS in the presence of obstructive jaundice was studied by determination of the survival rate of rats subjected to biliary obstruction and by polarographic determination of the hepatic mitochondrial function. Treatment with STS was found to result in a significant improvement in rats' survival rate ($p < 0.05$). Polarography demonstrated significant preservation of mitochondrial respiratory capacity after treatment with STS. The results of the present study show that the deterioration in liver function in the presence of biliary obstruction is probably caused by impairment of mitochondrial respiration. This may be preserved by treatment with STS. The exact explanation of its effect is not yet clear.

KEY WORDS: Biliary obstruction liver mitochondrial function sodium thiosulfate

### INTRODUCTION

Development of hepatic failure in the presence of longstanding obstructive jaundice, its pathophysiology and possible prevention still focuses the attention of surgeons and other specialists. It is well accepted today that biliary obstruction leads to a disturbance in liver mitochondrial function[1–4]. Sodium thiosulfate (STS) is an antidote used in cyanide poisoning by converting cyanide to inactive thiocyanate[5]. Recently, it was shown to have a protective effect on liver and renal functions by simultaneous administration with cisplatin[6–9].

In the present study we investigated the effect of STS administration on the survival rate and liver mitochondrial respiratory function in rats subjected to biliary obstruction.

### MATERIAL AND METHODS

*Animals*: In all experiments male Wistar rats weighing 200–250 g were used. The rats were fed on water and food *ad libitum*, before and after operation.
*Operations*: They were performed using ether anesthesia. Through a midline incision the bile duct was identified and ligated with 3/0 silk. In approximately 10–15% of the rats accessory bile ducts were identified and ligated. The abdomen was closed in layers using 4/0 Dexon$^R$. In the sham operated group opening and closure of the abdominal cavity were performed. After operation the rats were allocated to the following groups:

Group 1: Sham operated rats ($n = 10$);
Group 2: Bile duct ligation (BDL) ($n = 76$);
Group 3: BDL + STS treatment ($n = 114$).

Only rats which developed progressive hyperbilirubinemia were analysed. Sodium thiosulfate was administered intramuscularly in daily doses of 50 mg/kg, five times a week during two weeks. Administration of STS started 14 days after BDL.

### *Mitochondrial Respiratory Function*

Mitochondrial respiratory function assay was performed in group 1 (n = 10 rats), group 2 (n = 12 rats) and group 3 (n = 34 rats)[6] four weeks after BDL or sham operation.

The livers were removed and placed in ice cold manitol-sucrose solution (0.225 M manitol, 0.075 M sucrose). Mitochondria were fractioned by the method of Hogeboom and Schneider[10]. Mitochondrial respiratory function was measured polarographically with the oxygen consumption meter at 20°C temperature[2,11]. The respiratory control ratio, respiratory stimulation adenosine diphosphate/oxygen ratio (ADP/O), oxygen consumption rate of state 3 respiration, oxygen consumption rate after stimulation with dinitrophenol (DNP) were calculated from the mitochondrial respiratory curve.

Statistical analysis was performed according to the Students' $t$-test.

### RESULTS

Following BDL progressive hyperbilirubinemia developed. The mortality began in Group 2 (BDL only) on the third week after BDL and in Group 3 (BDL + STS treatment) on the fourth week after BDL. Survival rates in Group 3 were significantly higher during the fourth to the tenth week after BDL as compared with the group not treated with STS (Fig. 1).

Figure 2 illustrates schematically the mitochondrial respiration curve in the various studied groups. Administration of α-ketoglutarate as electron donor substrate resulted in mitochondrial respiration and reached a plateau in all 3 studied groups ($S_2$). Activation of phosphorylation by ADP administration caused a regulated respiratory stimulation in Group 1 and Group 3 which reached a plateau ($S_3$) and subsequently decreased to the prestimulation level ($S_4$). In contrast, the mitochondrial respiration of Group 2 following administration of ADP demonstrated only a slight unregulated increase in mitochondrial respir-

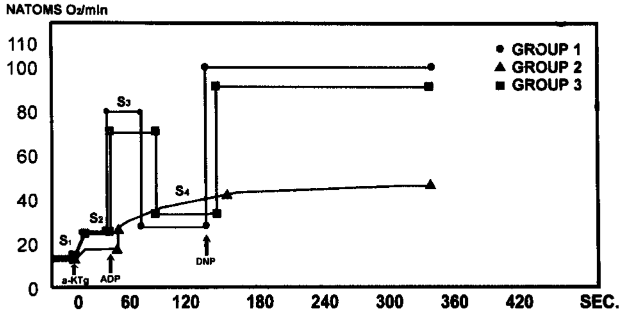

**Figure 2** Schematic presentation of mitochondrial respiratory curves in the 3 studied groups. In contrast to group 1 (sham operation) and 3 (BDL + STS), group 2 (BDL) demonstrates loss of regulation of mitochondrial respiratory function. Treatment with STS preserves the regulation of the mitochondrial respiratory function. $S_1$ and $S_4$ = rest periods of mitochondrial respiration; $S_2$ and $S_3$ = respiratory regulated plateau after stimulation; α-ktg = alfa ketoglutarate; ADP = adenosine diphosphate; DNP = dinitrophenol.

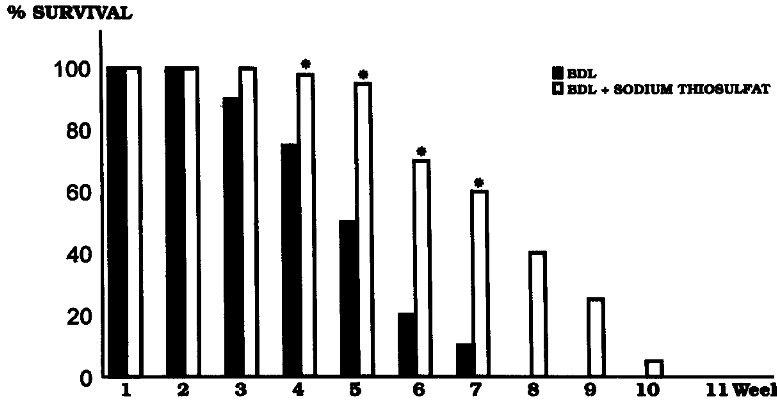

**Figure 1** Survival of rats subjected to BDL (group 2) and BDL + STS treatment (group 3). From the 4th to the 7th week post BDL survival rate was significantly higher in group 3. From the 8th to the 10th week post BDL only rats treated with STS survived (*p < 0.05).

ation which did not reach a plateau and did not decrease subsequently to the prestimulation level. Administration of dinitrophenol (DNP), an uncoupler of the oxidative phosphorylation (stimulates respiration of the mitochondria, but prevents formation of ATP) resulted in substantial stimulation of the mitochondrial respiration in Group 1 and 3, but not in Group 2. The various calculated parameters of mitochondrial respiration function are summarized in Table 1. It can be clearly seen that treatment with STS preserved most of the mitochondrial respiratory functions in rats subjected to obstructive jaundice of 3-weeks duration.

### DISCUSSION

The exact cause of liver failure occurring as a result of longstanding biliary obstruction is not yet clearly defined. Impaired mitochondrial respiratory function was observed during obstructive jaundice[1,2,4], and in other pathological states such as hemorrhagic and endotoxin shock[12-15]. The defective mitochondrial function is manifested by uncontrolled respiration or uncoupling of the respiration from phosphorylation.

Koyama *et al.*[1] found experimentally that relief of biliary obstruction at an early stage resulted in gradual improvement of mitochondrial functioning although it occurred more slowly than the improvement of the standard liver function tests. Clinically in patients with short-term biliary obstruction mitochondrial respiratory function was slightly impaired and showed marked improvement after relief of obstruction. On the contrary, in patients with long-term obstruction, severe mitochondrial respiratory dysfunction was evident which improved only slightly, 5–6 weeks after surgical release of the obstruction. According to the investigations of Koyama *et al.*[1,2] the mitochondrial damage in obstructive jaundice is nonspecific and may be attributed to high level of bile acid, especially chenodeoxycholic acid (CDCA). Zetterstrom and Ernster[4] postulated that uncoupling of oxidative phosphorylation is caused by bilirubin.

In our experimental study, impairment of liver mitochondrial function was demonstrated in biliary obstruction. In contrast to other reports concerning mitochondrial respiration in the presence of obstructive jaundice[1,2,4], our study demonstrates loss of regulation of mitochondrial respiration.

In recent publications, STS has been shown to be effective against cisplatinum toxicity. During administration of cisplatinum in combination with STS either parenterally or infused directly into the hepatic artery, effective protection against cisplatinum hepatic and renal toxicity was achieved[6-9]. These reports warrant the hypothesis that STS might have a beneficial effect on liver mitochondrial function during biliary obstruction.

The results of our study indicate that STS treatment increased significantly the survival rate of rats subjected to biliary obstruction. Liver mitochondrial function in the group treated with STS was mostly preserved due to regulation of mitochondrial respiration. Polarographic determination of these functions in the different studied groups was performed only once, four weeks after biliary obstruction, because the main goal of this study was the conceptual determination of the effect of STS. In future studies we shall try to determine the exact duration of the period in which STS is effective.

**Table 1** Various mitochondrial respiratory parameters in the 3 studied groups

| *Parameters** | *Group 1* | *Group 2* | *Group 3* |
|---|---|---|---|
| Respiratory control | $2.60 \pm 0.10$ | $1.00 \pm 0.08^{+}$ | $1.87 \pm 0.11^{++}$ |
| Respiratory stimulation | $3.13 \pm 0.21$ | $2.23 \pm 0.29^{+}$ | $2.69 \pm 0.20^{++}$ |
| Oxygen consumption rate in $S_3$ (n atoms $0_2$/min) | $78.30 \pm 3.35$ | $48.90 \pm 4.65^{+}$ | $69.40 \pm 6.12^{++}$ |
| ADP/O | $1.98 \pm 0.10$ | –** | $1.99 \pm 0.09^{++}$ |
| Oxygen consumption rate with succinate + glutamate + DNP | $100.80 \pm 8.7$ | $51.60 \pm 9.37^{+}$ | $93.30 \pm 9.51^{++}$ |
| Oxygen consumption rate with succinate + DNP | $100.30 \pm 9.92$ | $39.70 \pm 10.81^{+}$ | $91.10 \pm 8.34^{++}$ |

* Values are mean ± S. D.; DNP = dinitrophenol; ** Value impossible to calculate because of the lack of regulation;
$+ p < 0.01$ group 2 vs group 1; $^{++} p < 0.01$ group 3 vs group 2.

We could not yet give an exact explanation for the observed beneficial effect of STS. Possible explanations are: 1. Inhibitory effect of STS on bile acids and/ or bilirubin which may be responsible for suppression of liver mitochondrial function; 2. Stimulatory effect of STS on enzymatic processes which are involved in mitochondrial respiration. Although the results obtained in our study are promising, additional experimental and clinical investigations are needed for the determination of the exact role of STS in patients suffering from biliary obstruction. STS might be proved in the future to be effective in the extension of the biliary obstruction period after which complete recovery of liver dysfunction could be obtained.

### REFERENCES

1. Koyama, K., Takagi, Y., Ito, K. and Sato, T. (1981) Experimental and clinical studies on the effect of biliary drainage in obstructive jaundice. *Am. J. Surg.*, **142**, 293–9.
2. Koyama, K., Ito, K., Ouchi, K., Matsubara, S. and Sato, T. (1980) Mitochondrial function of rat liver in biliary obstruction. *Tohoku J. Exp. Med.*, **131**, 59–69.
3. Okawa, K., Takasan, H., Kitomava, O. *et al.* (1973) Alteration of liver mitochondrial metabolism in a patient with biliary obstruction due to liver carcinoma. *Am. J. Surg.*, **126**, 653–7.
4. Zetterstrom, R. and Ernster, L. (1956) Bilirubin, an uncoupler of oxidative phosphorylation in isolated mitochondria. *Nature*, **178**, 1335–7.
5. Reynolds, J. E. F. ed. Martindale. (1989) *The Extra Pharmacopoeia.* 29th ed. London: The Pharmaceutical Press, 855.
6. Abe, R., Akiyoshi, T., Koba, F., Tsuji, H. and Baba, T. (1988) "Two-route chemotherapy" using intraarterial cisplatin and intravenous sodium thiosulphate, its neutralizing agent, for hepatic malignancies. *Eur. J. Cancer Clin. Oncol.*, **24**, 1671–4.
7. Wagner, T., Kreft, B., Bohlmann, G. and Schnieder, G. (1988) Effects of fosfomycin, mesna and sodium thiosulphate on the toxicity and antitumor activity of cisplatin. *J. Cancer Res. Clin. Oncol.*, **114**, 497–501.
8. Onohava, S., Kobayashi, H., Itoh, Y. and Shinohava, S. (1988) Intraarterial cisplatinum infusion with sodium thiosulphate protection and angiotensin II induced hypertension for treatment of hepatocellular carcinoma. *Acta. Radiol.*, **29**, 197–202.
9. Abe, R., Akiyoshi, T. and Baba, T. (1990) Inactivation of cis-diamminedichloroplatinum (II) in blood by sodium thiosulphate. *Oncology*, **47**, 65–9.
10. Hogeboom, G. H. and Schreider, W. C. (1948) Isolation of intact mitochondria from rat liver. *J. Biol. Chem.*, **172**, 619–30.
11. Hagihava, B. (1961) Techniques for the application of polarography to mitochondrial respiration. *Biochim. Biophys. Acta.*, **46**, 134–42.
12. Mela, L., Bacako, L. V. and Miller, L. D. (1971) Defective oxidative metabolism of rat liver mitochondria in haemorrhage and endotoxin shock. *Am. J. Physiol.*, **220**, 571–7.
13. Hift, H. and Starwitz, J. G. (1961) Irreversible hemorrhagic shock in dogs: structure and function of liver mitochondria. *Am. J. Physiol.*, **200**, 264–8.
14. Moss, G. S., Erre, P. P. and Schumer, W. (1969) Effect of endotoxin on mitochondrial respiration. *Surg. Forum.*, **20**, 24–6.
15. Schumer, W., Das Gupta, T. K., Moss, G. S. and Myhus, L. M. (1970) Effect of endotoxemia on liver cell mitochondria in man. *Ann. Surg.*, **171**, 875–82.

